# Supplementary material for: The differential effect of optogenetic serotonergic manipulation on sustained motor actions and waiting for future rewards in mice
Source: Front Neurosci. 2024 Sep 25;18:1433061. doi: 10.3389/fnins.2024.1433061 (PMC11461476; doi:10.3389/fnins.2024.1433061)
Supplement: Supplementary file 1 [file Data_Sheet_1.docx]

**Table S1. Statistical details for optogenetic activation study**

| Fig. | Sample size | Mean and SEM | Statistical test | Values |
| --- | --- | --- | --- | --- |
| 3B | 8 ChR2 mice | Yellow: 14.99 ± 0.37 s  Blue: 17.39 ± 0.49 s | paired t-test | *t*_7_ = 6.31, p = 0.00040 |
| 3C | 5 control mice | Yellow: 14.63 ± 0.74 s  Blue: 14.45 ± 0.68 s | paired t-test | *t*_4_ = 1.50, p = 0.21 |
| 3D | 8 ChR2 mice 5 control mice | ChR2: 0.16 ± 0.027 Control: -0.012 ± 0.0087 | unpaired t-test | *t*_11_ = 4.98, p = 0.00042 |
| 4A | 8 ChR2 mice | 64 press yellow:  95.02 ± 1.73%  64 press blue:  94.55 ± 3.23% | paired t-test | *t*_7_ = 0.23, p = 0.83 |
| 4B | 5 control mice | 64 press yellow:  90.08 ± 3.46%  64 press blue:  90.33 ± 2.86% | paired t-test | *t*_4_ = 0.073, p = 0.95 |
| 4D(i) | 8 ChR2 mice | Yellow: 48.68 ± 3.09 s Blue: 48.86 ± 2.27 s | paired t-test | *t*_7_ = 0.083, p = 0.94 |
| 4D(ii) | 8 ChR2 mice | Yellow: 111.50 ± 3.09 Blue: 108.63 ± 3.74 | paired t-test | *t*_7_ = 0.93, p = 0.38 |
| 4D(iii) | 8 ChR2 mice | Yellow: 15.41 ± 3.53 s Blue: 19.03 ± 6.37 s | signed-rank test | z = 1.26, p = 0.23 |
| 4E(i) | 5 control mice | Yellow: 43.86 ± 3.39 s Blue: 43.92 ± 3.73 s | paired t-test | *t*_4_ = 0.026, p = 0.98 |
| 4E(ii) | 5 control mice | Yellow: 115.2 ± 7.55 Blue: 112.2 ± 3.74 | paired t-test | *t*_4_ = 0.58, p = 0.59 |
| 4E(iii) | 5 control mice | Yellow: 11.24 ± 1.75 s Blue: 10.09 ± 1.10 s | paired t-test | *t*_4_ = 1.11, p = 0.33 |
| 4F(i) | 8 ChR2 mice 5 control mice | ChR2: 0.016 ± 0.042 Control: 0.0048 ± 0.0052 | unpaired t-test | *t*_11_ = 0.16, p = 0.88 |
| 4F(ii) | 8 ChR2 mice 5 control mice | ChR2: -0.024 ± 0.029 Control: -0.016 ± 0.041 | unpaired t-test | *t*_11_ = 0.17, p = 0.87 |
| 4F(iii) | 8 ChR2 mice 5 control mice | ChR2:0.13 ± 0.090 Control: -0.059 ± 0.096 | unpaired t-test | *t*_11_ = 1.40, p = 0.19 |
| 5B | 8 ChR2 mice | Yellow: 16.94 ± 2.65 s Blue: 19.80 ± 3.70 s | signed-rank test | z = 1.26, p = 0.23 |
| 5C | 5 control mice | Yellow: 10.56 ± 1.47 s Blue: 11.92 ± 1.06 s | paired t-test | *t*_4_ = 1.61, p = 0.18 |
| 5D | 8 ChR2 mice 5 control mice | ChR2: 0.17 ± 0.11 Control: 0.17 ± 0.096 | unpaired t-test | *t*_11_ = 0.0027, p = 0.998 |

**Table S2. Details of repeated measures ANOVA analysis of short IPIs in the optogenetic activation studies**

| Sample size | Factors | Values |
| --- | --- | --- |
| 8 ChR2 mice | Stimulation (2 level, within) Press (5 level, within) | Stimulation: *F*(1,7) = 5.55, *P* = 0.051  Press: *F*(4,28) = 25.64, *P* = 5.2 x 10^-9^  Interaction: *F*(4,28) = 1.86, *P* = 0.15 |
| 5 control mice | Stimulation (2 level, within) Press (5 level, within) | Stimulation: *F*(1,4) = 0.16, *P* = 0.71  Press: *F*(4,16) = 42.62, *P* = 2.4 x 10^-8^  Interaction: *F*(4,16) = 0.29, *P* = 0.88 |

**Table S3. Repeated measures ANOVA on optogenetic activation studies**

| Behavioral measures | Sample size | Factors | Values |
| --- | --- | --- | --- |
| The number of lever-presses | 8 ChR2 and  5 Control mice | Stimulation (2 level, within)  Genotype (2 level, between) | Genotype: *F*(1,11) = 0.41, *P* = 0.54  Stimulation: *F*(1,11) = 1.10, *P* = 0.32  Interaction: *F*(1,11) = , *P* = 0.98 |
| Time spent lever-pressing |  |  | Genotype: *F*(1,11) = 1.41, *P* = 0.26  Stimulation: *F*(1,11) = 0.005, *P* = 0.94  Interaction: *F*(1,11) = 0.001, *P* = 0.97 |
| Long IPI |  |  | Genotype: *F*(1,11) = 3.36, *P* = 0.94  Stimulation: *F*(1,11) = 1.51, *P* = 0.25  Interaction: *F*(1,11) = 0.19, *P* = 0.67 |
| Short IPI |  | Stimulation (2 level, within)  Press (5 level, within)  Genotype (2 level, between) | Genotype: *F*(1,9) = 2.12, *P* = 0.18  Stimulation: *F*(1,11) = 2.12, *P* = 0.089  Press: *F*(4,44) = 30.93, *P* = 0.00019  Genotype x Stimulation x Press:  *F*(4,44) = 0.36, *P* = 0.84  Genotype x Stimulation:  *F*(1,44) = 1.91, *P* = 0.19  Genotype x Press:  *F*(4,44) = 5.02, *P* = 0.0020  Stimulation x Press:  *F*(4,44) = 1.32, *P* = 0.28 |

**
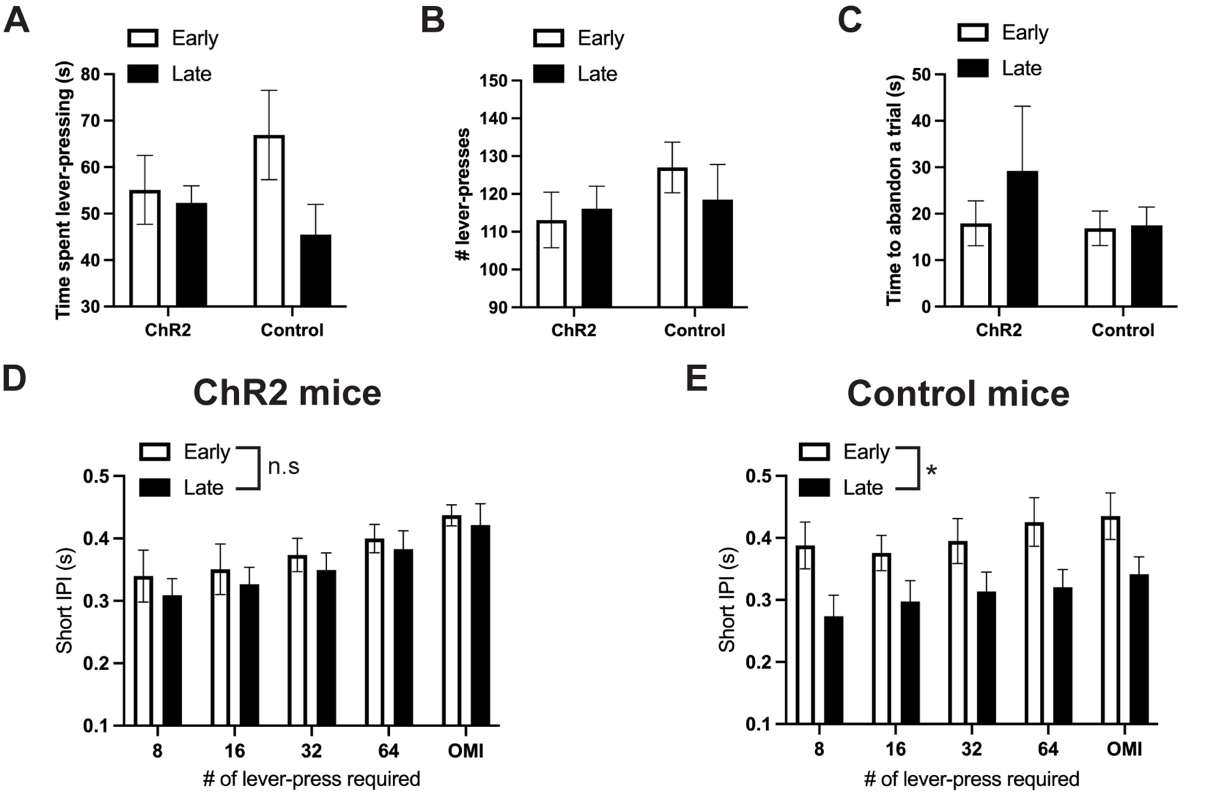
**

**Figure S1. Repeated optogenetic activation did not remarkably change motor actions in control trials across testing sessions.** Each bar indicates the mean of behavioral measures in the first 2 (Early, white) or last 2 (Late, black) sessions. (A) Time spent lever-pressing in omission trials. (B) The number of lever-presses in omission. (C) The time to abandon a trial. (D) Short IPIs in ChR2 mice and (E) in control mice. Error bars represent the SEM in all graphs. n.s. p > 0.05, * < 0.05. See Table S4 for the detail of statistical tests.

**Table S4. Statistical Details for Fig. S1**

| Behavioral measures | Sample size | Factors | Values |
| --- | --- | --- | --- |
| Time spent lever-pressing  (Fig. S1A) | 8 ChR2 and  5 Control mice | Session (2 level, within)  Genotype (2 level, between) | Session: *F*(1,11) = 5.10, *P* = 0.045  Genotype: *F*(1,11) = 0.092, *P* = 0.77  Interaction: *F*(1,11) = 3.03, *P* = 0.11 |
| The number of lever-presses  (Fig. S1B) |  |  | Session: *F*(1,11) = 0.16, *P* = 0.70  Genotype: *F*(1,11) = 1.04, *P* = 0.33  Interaction: *F*(1,11) = 0.67, *P* = 0.43 |
| Time to abandon a trial  (Fig. S1C) |  |  | Session: *F*(1,11) = 0.85, *P* = 0.38  Genotype: *F*(1,11) = 0.28, *P* = 0.61  Interaction: *F*(1,11) = 0.68, *P* = 0.43 |
| Short IPI  (Fig. S1D & E) | 8 ChR2 mice | Session (2 level, within) Press (5 level, within) | Session: *F*(1,7) = 1.54, *P* = 0.25  Press: *F*(4,12) = 20.19, *P* = 6.4 x 10^-8^  Interaction: *F*(4,28) = 0.14, *P* = 0.97 |
|  | 5 control mice | Session (2 level, within) Press (5 level, within) | Session: *F*(1,4) = 15.36, *P* = 0.017  Press: *F*(4,16) = 12.16, *P* = 9.9 x 10^-5^  Interaction: *F*(4,16) = 1.33, *P* = 0.30 |


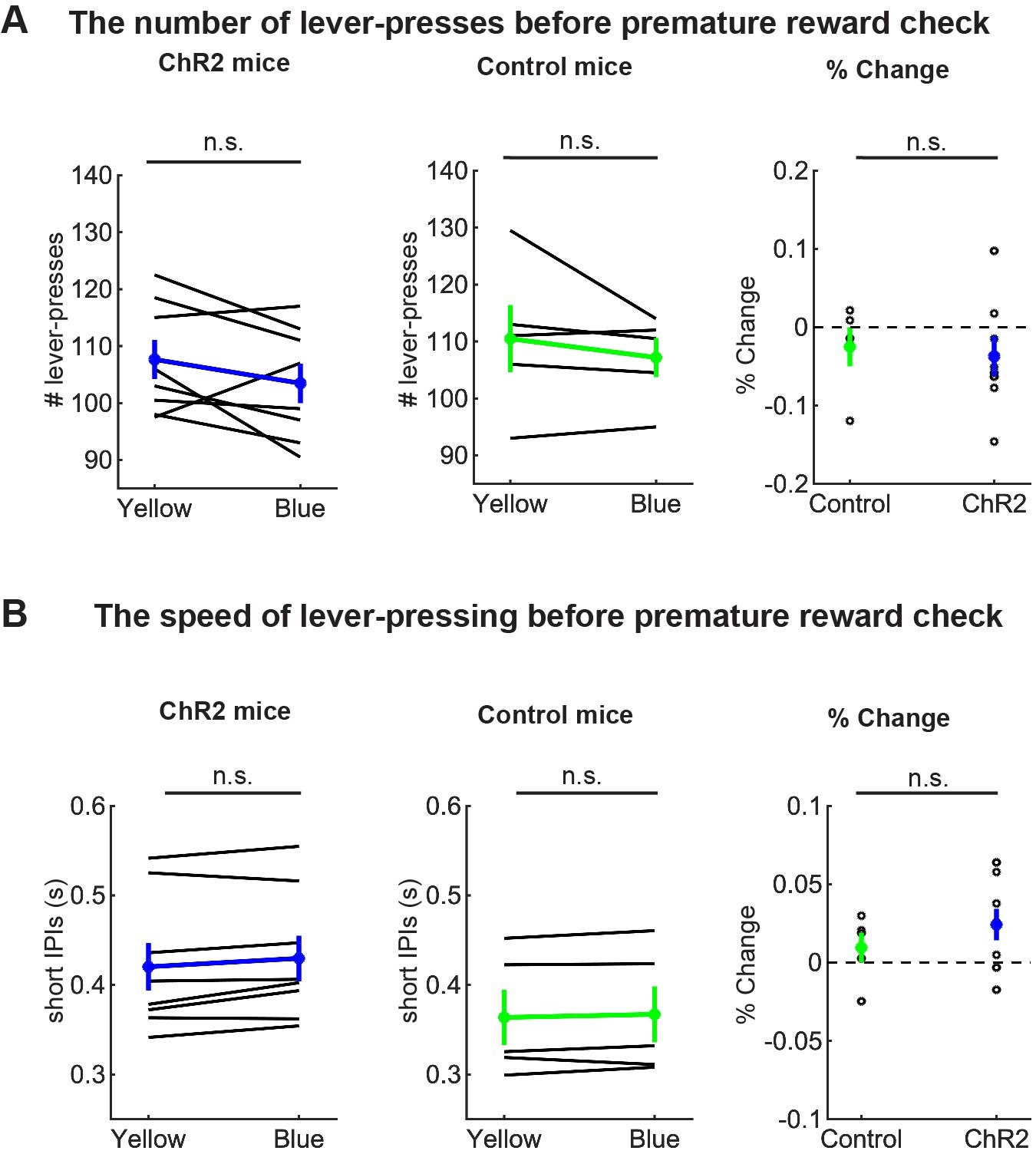


**Figure S2. Optogenetic activation did not change the summary statistics of action persistence and speed before premature reward check in summary statistics** (A) The number of lever-presses before premature reward check in omission trials in ChR2 (Left, *n* = 8 mice) and control (Middle, *n* = 5 mice) mice. Blue and green dots indicate the means across ChR2 and control mice data, respectively. Right panel indicates change of the number of lever-presses in blue light trials to yellow light trials in control (*n* = 5 mice) and ChR2 (*n* = 8 mice) mice. Green and blue-filled circles indicate the means across control and ChR2 mice, respectively. (B) Short IPIs before premature reward check in omission trials in ChR2 (Left, *n* = 8 mice) and control (Middle, *n* = 5 mice) mice. Blue and green dots indicate the means across ChR2 and control mice data, respectively. Right panel indicates the change of the short IPIs in blue light trials to yellow light trials in control (*n* = 5 mice) and ChR2 (*n* = 8 mice) mice. Green- and blue-filled circles indicate the means across control and ChR2 mice, respectively. Error bars represent the SEM in all graphs. n.s. p > 0.05. See Table S5 for the detail of statistical tests.

**Table S5. Statistical details for Figure S2**

| Fig. | Sample size | Mean and SEM | Statistical test | Values |
| --- | --- | --- | --- | --- |
| S3A | 8 ChR2 mice | Yellow: 107.63 ± 3.44  Blue: 103.44 ± 3.49 | paired t-test | *t*_7_ = 1.56, p = 0.16 |
| S3A | 5 control mice | Yellow: 110.50 ± 5.89  Blue: 107.20 ± 3.44 | paired t-test | *t*_4_ = 1.05, p = 0.21 |
| S3A | 8 ChR2 mice  5 control mice | ChR2: -0.037 ± 0.026  Control: -0.025 ± 0.025 | unpaired t-test | *t*_11_ = 0.31, p = 0.76 |
| S3B | 8 ChR2 mice | Yellow: 0.42 ± 0.027 s  Blue: 0.43 ± 0.026 s | paired t-test | *t*_7_ = 2.32, p = 0.053 |
| S3B | 5 control mice | Yellow: 0.36 ± 0.031 s  Blue: 0.37 ± 0.032 s | paired t-test | *t*_4_ = 1.10, p = 0.33 |
| S3B | 8 ChR2 mice  5 control mice | ChR2: 0.024 ± 0.010  Control: 0.0095 ± 0.0096 | paired t-test | *t*_7_ = 0.99, p = 0.35 |

**Table S6. GLM analysis of the number of lever-press in the optogenetic activation study**

| Group | Effect | Estimate | 95% CI  lower / upper | t-stats | p-values |
| --- | --- | --- | --- | --- | --- |
| ChR2 | Intercept | 4.70 | 4.66 / 4.75 | *t*_611_ = 197.18 | 0 |
|  | Manipulation | -0.026 | -0.041 / -0.011 | *t*_611_ = 3.38 | 0.00077 |
|  | Elapsed time | −3*.*2×10^−5^ | −4*.*0×10^−5^ / −2*.*4×10^−5^ | *t*_611_ = 7.5 | 2*.*2×10^−13^ |
|  | Sessions | 0.0095 | 0.0060 / 0.0130 | *t*_611_ = 5.3 | 1*.*6×10^−7^ |
| Control | Intercept | 4.73 | 4.68 / 4.78 | *t*_390_ = 169.82 | 0 |
|  | Manipulation | -0.0057 | -0.024 / 0.013 | *t*_390_ = 0.60 | 0.55 |
|  | Elapsed time | −4*.*5×10^−5^ | −5*.*6×10^−5^ / −3*.*4×10^−5^ | *t*_390_ = 8.22 | 3*.*0×10^−15^ |
|  | Sessions | 0.012 | 0.0074 / 0.016 | *t*_390_ = 5.40 | 1*.*1×10^−7^ |

**Table S7. GLM analysis of short IPIs in the optogenetic activation studies**

| Group | Effect | Estimate | 95% CI  lower / upper | t-stats | p-values |
| --- | --- | --- | --- | --- | --- |
| ChR2 | Intercept | 0.470 | 0.42 / 0.52 | *t*_611_ = 19.34 | 2*.*4×10^−65^ |
|  | Manipulation | 0.012 | 0.0021 / 0.021 | *t*_611_ = 2.4 | 0.017 |
|  | Elapsed time | −1*.*2×10^−5^ | −1*.*7×10^−5^ / −6*.*7×10^−6^ | *t*_611_ = 4.5 | 6*.*9×10^−6^ |
|  | Sessions | -0.0064 | -0.0085 / -0.0042 | *t*_611_ = 5.75 | 1*.*3×10^−8^ |
| Control | Intercept | 0.46 | 0.40 / 0.52 | *t*_390_ = 15.80 | 9*.*0×10^−44^ |
|  | Manipulation | 0.0028 | -0.0056 / 0.011 | *t*_390_ = 0.65 | 0.52 |
|  | Elapsed time | −6*.*6×10^−6^ | −1*.*1×10^−5^ / −1*.*8×10^−6^ | *t*_390_ = 2.69 | 0.0074 |
|  | Sessions | -0.018 | -0.020 / -0.016 | *t*_390_ = 18.61 | 9*.*2×10^−56^ |

**Table S8. Statistical details of the optogenetic inhibition studies**

| Fig | Sample size | Mean and SEM | Statistical test | Values |
| --- | --- | --- | --- | --- |
| 7B | 5 ArchT mice | No light: 15.50 ± 0.88 s Yellow: 14.11 ± 0.96 s | paired t-test | *t*_4_ = 10.27, p = 0.00051 |
| 7C | 4 control mice | No light: 16.86 ± 0.73 s Yellow: 17.04 ± 0.66 s | paired t-test | *t*_3_ = 0.43, p = 0.70 |
| 7D | 5 ArchT mice 4 control mice | ArchT: -0.092 ± 0.013 Control: 0.012 ± 0.024 | unpaired t-test | *t*_7_ = 4.07, p = 0.0047 |
| 8A | 6 ArchT mice | 64 press no light:  99.24 ± 0.76%  64 press yellow:  98.89 ± 1.11% | paired t-test | *t*_5_ = 0.24, p = 0.82 |
| 8B | 5 control mice | 64 press no-light:  100 ± 0%  64 press yellow:  99.05 ± 0.95% | paired t-test | *t*_4_ = 1.0, p = 0.37 |
| 8D(i) | 6 ArchT mice | No light: 45.19 ± 1.97 s Yellow: 47.25 ± 2.47 s | signed-rank test | W = 4.0, p = 0.22 |
| 8D(ii) | 6 ArchT mice | No light: 117.57 ± 4.54 Yellow: 118.01 ± 4.85 | signed-rank test | W = 10, p = 1.0 |
| 8D(iii) | 6 ArchT mice | No light: 11.14 ± 1.05 s Yellow: 9.51 ± 0.82 s | paired t-test | *t*_5_ = 3.69, p = 0.014 |
| 8E(i) | 5 control mice | No light: 59.41 ± 5.20 s Yellow: 61.82 ± 5.44 s | paired t-test | *t*_4_ = 1.33, p = 0.25 |
| 8E(ii) | 5 control mice | No light: 123.48 ± 7.53 Yellow: 128.65 ± 6.17 | paired t-test | *t*_4_ = 2.03, p = 0.11 |
| 8E(iii) | 5 control mice | No light: 12.60 ± 2.21 s Yellow: 15.05 ± 3.47 s | paired t-test | *t*_4_ = 1.77, p = 0.15 |
| 8F(i) | 6 ArchT mice 5 control mice | ArchT: 0.046 ± 0.030 Control: 0.041 ± 0.030 | unpaired t-test | *t*_9_ = 0.11, p = 0.91 |
| 8F(ii) | 6 ArchT mice 5 control mice | ArchT: 0.0056 ± 0.029 Control: 0.046 ± 0.023 | unpaired t-test | *t*_9_ = 1.07, p = 0.31 |
| 8F(iii) | 6 ArchT mice 5 control mice | ArchT: -0.14 ± 0.031 Control: 0.16 ± 0.076 | unpaired t-test | *t*_9_ = 3.93, p = 0.0034 |
| 9B | 6 ArchT mice | No light: 13.50 ± 1.52 s Yellow: 20.31 ± 7.40 s | signed-rank test | W = 7.0, p = 0.56 |
| 9C | 5 control mice | No light: 18.50 ± 2.82 s Yellow: 16.42 ± 3.76 s | paired t-test | *t*_4_ = 0.92, p = 0.41 |
| 9D | 6 ArchT mice 5 control mice | ArchT: 0.37 ± 0.34 Control: -0.11 ± 0.14 | U test | *U* = 21.0, p = 0.33 |

**Table S9. Details of repeated measures ANOVA analysis of short IPIs in the optogenetic inhibition studies**

| Sample size | Factors | Values |
| --- | --- | --- |
| 6 ArchT mice | Stimulation (2 level, within) Press (5 level, within) | Stimulation: *F*(1,5) = 0.28, *P* = 0.87  Press: *F*(4,20) = 1.86, *P* = 0.16  Interaction: *F*(4,20) = 2.53, *P* = 0.073 |
| 5 control mice | Stimulation (2 level, within) Press (5 level, within) | Stimulation: *F*(1,4) = 0.031, *P* = 0.87  Press: *F*(4,16) = 1.27, *P* = 0.32  Interaction: *F*(4,16) = 0.37, *P* = 0.83 |

**Table S10. Repeated measures ANOVA on optogenetic inhibition studies**

| Behavioral measures | Sample size | Factors | Values |
| --- | --- | --- | --- |
| The number of lever-presses | 6 ArchT and  5 Control mice | Stimulation (2 level, within)  Genotype (2 level, between) | Genotype: *F*(1,9) = 1.13, *P* = 0.32  Stimulation: *F*(1,9) = 1.67, *P* = 0.23  Interaction: *F*(1,9) = 1.19, *P* = 0.30 |
| Time spent lever-pressing |  |  | Genotype: *F*(1,9) = 7.42, *P* = 0.023  Stimulation: *F*(1,9) = 3.95, *P* = 0.078  Interaction: *F*(1,9) = 0.024, *P* = 0.88 |
| Long IPI |  |  | Genotype: *F*(1,9) = 0.010, *P* = 0.92  Stimulation: *F*(1,9) = 0.43, *P* = 0.53  Interaction: *F*(1,9) = 1.51, *P* = 0.25 |
| Short IPI |  | Stimulation (2 level, within)  Press (5 level, within)  Genotype (2 level, between) | Genotype: *F*(1,9) = 2.12, *P* = 0.18  Stimulation: *F*(1,9) = 0.059, *P* = 0.81  Press: *F*(4,36) = 2.69, *P* = 0.046  Genotype x Stimulation x Press:  *F*(4,36) = 0.85, *P* = 0.50  Genotype x Stimulation:  *F*(1,36) = 0.00016, *P* = 0.99  Genotype x Press:  *F*(4,36) = 0.38, *P* = 0.82  Stimulation x Press:  *F*(4,36) = 0.66, *P* = 0.62 |

**
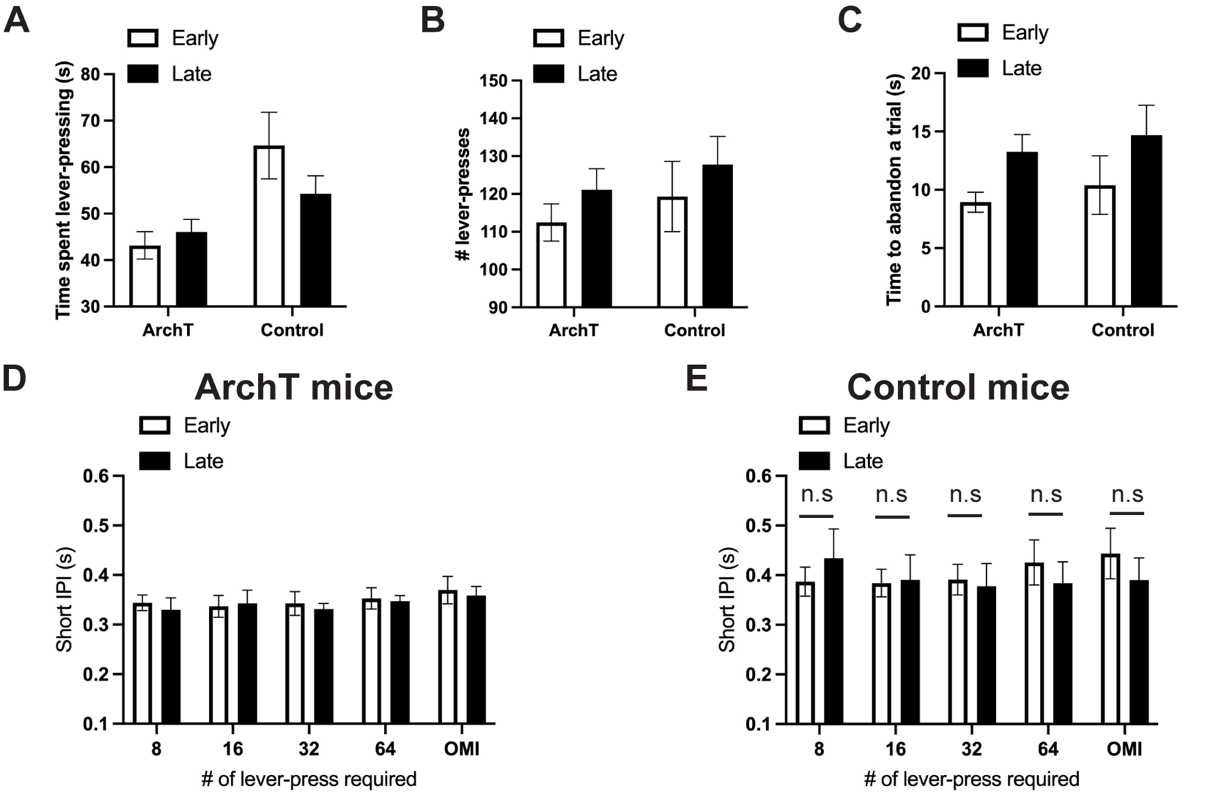
**

**Figure S3. Repeated optogenetic inhibition did not remarkably change motor actions in control trials across testing sessions.** Each bar indicates the mean of behavioral measures in the first 2 (Early, white) or last 2 (Late, black) sessions. (A) Time spent lever-pressing in omission trials. (B) The number of lever-presses in omission. (C) The time to abandon a trial. (D) Short IPIs in ArchT mice and (E) in control mice. Error bars represent the SEM in all graphs. n.s. p > 0.05. See Table S11 for the detail of statistical tests.

**Table S11. Statistical Details for Fig. S3**

| Behavioral measures | Sample size | Factors | Values |
| --- | --- | --- | --- |
| Time spent lever-pressing  (Fig. S3A) | 6 ArchT and  5 Control mice | Session (2 level, within)  Genotype (2 level, between) | Session: *F*(1,9) = 1.47, *P* = 0.26  Genotype: *F*(1,9) = 8.11, *P* = 0.019  Interaction: *F*(1,9) = 4.62, *P* = 0.060 |
| The number of lever-presses  (Fig. S3B) |  |  | Session: *F*(1,9) = 3.41, *P* = 0.098  Genotype: *F*(1,9) = 0.65, *P* = 0.44  Interaction: *F*(1,9) = 0.00050, *P* = 0.98 |
| Time to abandon a trial  (Fig. S3C) |  |  | Session: *F*(1,9) = 9.85, *P* = 0.012  Genotype: *F*(1,9) = 0.41, *P* = 0.54  Interaction: *F*(1,9) = 0.000090, *P* = 0.93 |
| Short IPI  (Fig. S3D & E) | 6 ArchT mice | Session (2 level, within)  Press (5 level, within) | Session: *F*(1,5) = 0.12, *P* = 0.75  Press: *F*(4,20) = 1.32, *P* = 0.30  Interaction: *F*(4,20) = 0.15, *P* = 0.96 |
|  | 5 control mice | Session (2 level, within)  Press (5 level, within) | Session: *F*(1,4) = 0.13, *P* = 0.74  Press: *F*(4,16) = 1.73, *P* = 0.19  Interaction: *F*(4,16) = 4.70, *P* = 0.011 |

**
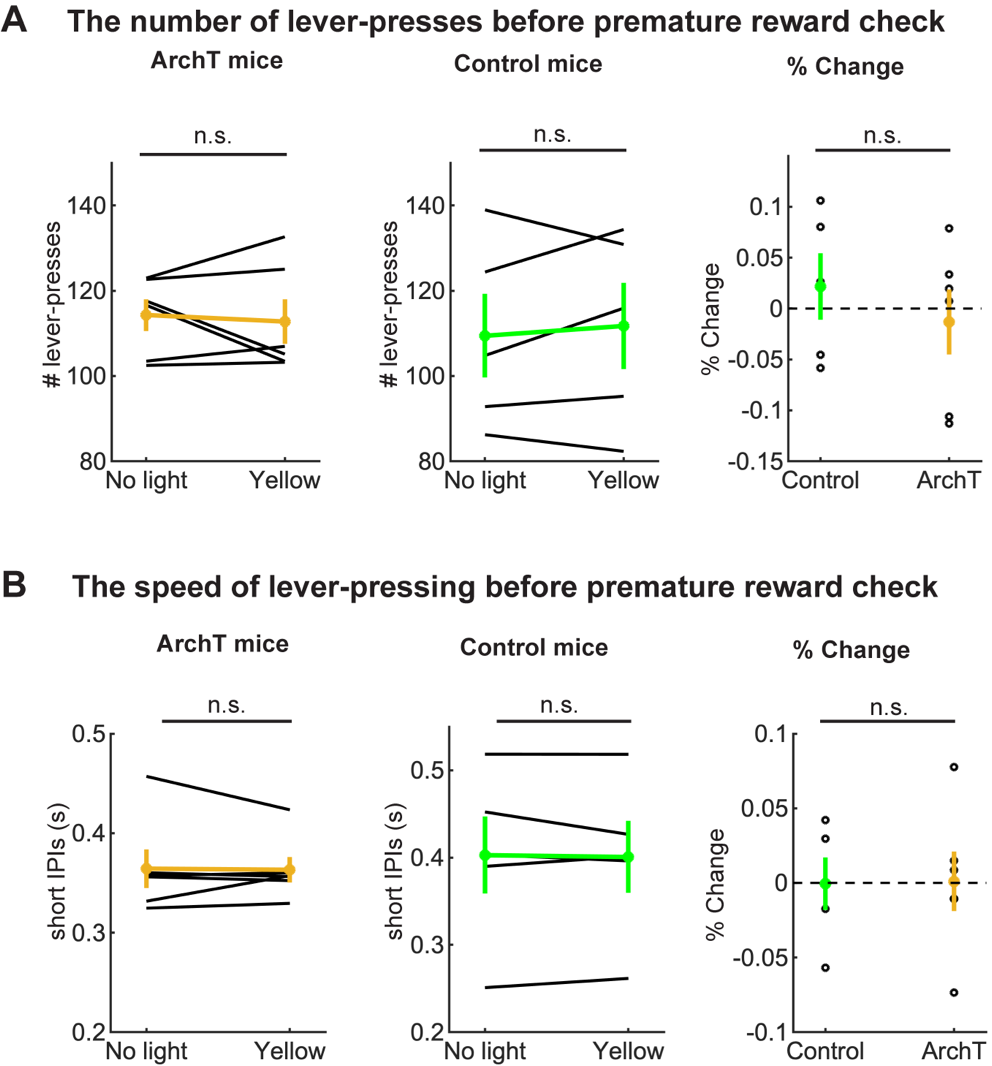
**

**Figure S4. Optogenetic inhibition did not change the summary statistics of action persistence and speed before premature reward check.** (A) The number of lever-presses before premature reward check in omission trials in ArchT (Left, *n* = 6 mice) and control (Middle, *n* = 5 mice) mice. Yellow and green dots indicate the means across ArchT and control mice, respectively. Right panel indicates change of the number of lever-presses in yellow light trials to no light trials in control (*n* = 5 mice) and ArchT (*n* = 6 mice) mice. Green- and yellow-filled circles indicate the means across control and ArchT mice, respectively. (B) Short IPIs before premature reward check in omission trials in ArchT (Left, *n* = 6 mice) and control (Middle, *n* = 5 mice) mice. Yellow and green dots indicate the means across ArchT and control mice, respectively. Right panel indicates the change of the short IPIs in yellow light trials to no light trials in control (*n* = 5 mice) and ArchT (*n* = 6 mice) mice. Green- and yellow-filled circles indicate the means across control and ArchT mice, respectively. Error bars represent the SEM in all graphs. n.s. p > 0.05. See Table S12 for the detail of statistical tests.

**Table S12. Statistical details for Figure S4**

| Fig. | Sample size | Mean and SEM | Statistical test | Values |
| --- | --- | --- | --- | --- |
| S4A | 6 ArchT mice | No light: 114.29 ± 3.73  Yellow: 112.74 ± 5.22 | signed-rank test | W = 10, p = 1.0 |
| S4A | 5 control mice | No light: 109.44 ± 9.83  Yellow: 111.74 ± 10.08 | paired t-test | *t*_4_ = 0.61, p = 0.57 |
| S4A | 6 ArchT mice 5 control mice | ArchT: -0.013 ± 0.032  Control: 0.022 ± 0.033 | unpaired t-test | *t*_9_ = 0.76, p = 0.47 |
| S4B | 6 ArchT mice | No light: 0.36 ± 0.020 s  Yellow: 0.36 ± 0.013 s | signed-rank test | W = 10, p = 1.0 |
| S4B | 5 control mice | No light: 0.40 ± 0.044 s  Yellow: 0.40 ± 0.041 s | paired t-test | *t*_4_ = 0.32, p = 0.77 |
| S4B | 6 ArchT mice 5 control mice | ArchT: 0.0011 ± 0.0200  Control: -5.6 x 10^-4^ ± 0.018 | paired t-test | *t*_9_ = 0.059, p = 0.95 |

**Table S13. generalized linear mixed effect model analysis of the number of lever-press in optogenetic inhibition studies**

| Group | Effect | Estimate | 95% CI  lower / upper | t-stats | p-values |
| --- | --- | --- | --- | --- | --- |
| ArchT | Intercept | 4.77 | 4.70 / 4.85 | *t*_227_ = 124.03 | 1*.*5×10^−210^ |
|  | Manipulation | -0.0044 | -0.029 / 0.020 | *t*_227_ = 0.36 | 0.72 |
|  | Elapsed time | −6*.*4×10^−5^ | −8*.*2×10^−5^ / −4*.*7×10^−5^ | *t*_227_ = 7.14 | 1*.*2×10^−11^ |
|  | Sessions | 0.011 | 0.00021 / 0.022 | *t*_227_ = 2.01 | 0.046 |
| Control | Intercept | 4.74 | 4.58 / 4.90 | *t*_203_ = 59.94 | 4*.*7×10^−131^ |
|  | Manipulation | 0.0172 | -0.00890 / 0.0433 | *t*_203_ = 1.30 | 0.20 |
|  | Elapsed time | −6*.*7×10^−5^ | −8*.*2×10^−5^ / −5*.*1×10^−5^ | *t*_203_ = 8.45 | 5*.*4×10^−15^ |
|  | Sessions | 0.017 | 0.0047 / 0.028 | *t*_203_ = 2.77 | 0.0061 |

**Table S14. generalized linear mixed effect model analysis of short IPIs in optogenetic inhibition studies**

| Group | Effect | Estimate | 95% CI  lower / upper | t-stats | p-values |
| --- | --- | --- | --- | --- | --- |
| ArchT | Intercept | 0.39 | 0.35 / 0.42 | *t*_227_ = 22.099 | 1*.*7×10^−58^ |
|  | Manipulation | -0.0026 | -0.015 / 0.0097 | *t*_227_ = 0.41 | 0.68 |
|  | Elapsed time | −5*.*8×10^−6^ | −1*.*5×10^−5^ /3*.*1×10^−6^ | *t*_227_ = 1.3 | 0.20 |
|  | Sessions | -0.0065 | -0.012 / -0.00090 | *t*_227_ = 2.3 | 0.023 |
| Control | Intercept | 0.49 | 0.41 / 0.57 | *t*_203_ = 11.66 | 0.57 |
|  | Manipulation | -0.0029 | -0.021 / 0.016 | *t*_203_ = 0.31 | 0.76 |
|  | Elapsed time | −2*.*1×10^−5^ | −3*.*2×10^−5^ / −1*.*1×10^−5^ | *t*_203_ = 3.92 | 0.00012 |
|  | Sessions | -0.021 | -0.029 / -0.013 | *t*_203_ = 4.94 | 1*.*6×10^−6^ |

**
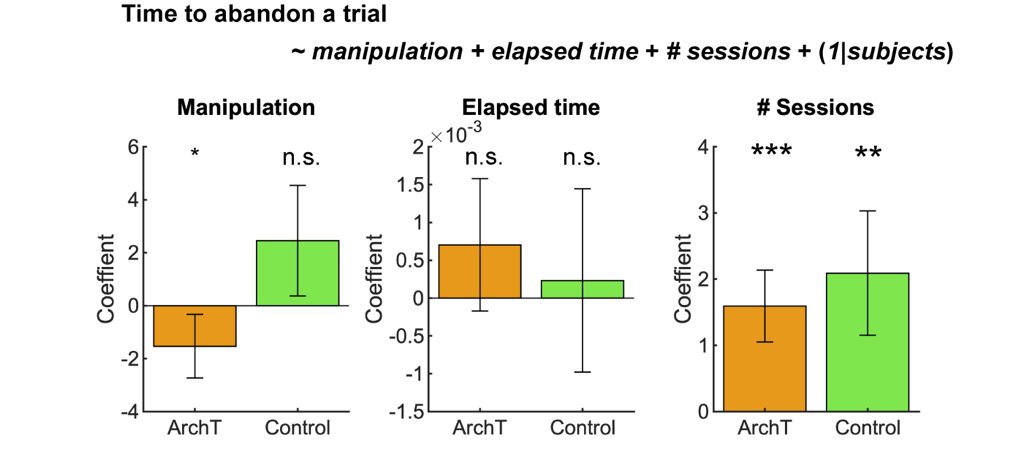
**

**Figure S5. GLM analysis showed the decrease in the time to abandon a trial by optogenetic inhibition consistent with the summary statistics analysis.** Orange and green bars indicate coefficients of optic manipulation (left), elapsed time (middle), and the number of sessions (right) in ArchT (*n* = 6 mice) and control (*n* = 5 mice) mice, respectively. Error bars indicate 95% confidence intervals of the coefficients. n.s. p > 0.05, * < 0.05, ** p < 0.01, *** p<0.001.

**Table S15. generalized linear mixed effect model analysis of the time to abandon a trial in optogenetic inhibition studies**

| Group | Effect | Estimate | 95% CI  lower / upper | t-stats | p-values |
| --- | --- | --- | --- | --- | --- |
| ArchT | Intercept | 0.019 | 0.015 / 0.024 | *t*_227_ = 8.88 | 2.0×10^−16^ |
|  | Manipulation | 0.0027 | 0.00035/ 0.0050 | *t*_227_ = 0.024 | 0.024 |
|  | Elapsed time | −1*.*3×10^−6^ | −2.9×10^−6^ /3.1×10^−7^ | *t*_227_ = 1.60 | 0.11 |
|  | Sessions | -0.0032 | -0.0043 / -0.0021 | *t*_227_ = 5.79 | 2.3×10^−8^ |
| Control | Intercept | 0.0099 | 0.0061 / 0.014 | *t*_203_ = 5.17 | 5.6×10^−7^ |
|  | Manipulation | -0.0017 | -0.0038 / 0.00032 | *t*_203_ = 1.66 | 0.098 |
|  | Elapsed time | −4.6×10^−7^ | −6.9×10^−5^ / 1.6×10^−5^ | *t*_203_ = 0.79 | 0.43 |
|  | Sessions | -0.0016 | -0.0025 / -0.00060 | *t*_203_ = 3.23 | 0.0014 |
